# Internal lead shielding for clinical electron treatments

**DOI:** 10.1002/acm2.14196

**Published:** 2023-11-03

**Authors:** Patrick N. McDermott

**Affiliations:** ^1^ Beaumont Health William Beaumont University Hospital Royal Oak Michigan USA

**Keywords:** backscatter, electrons, internal bolus, internal shielding

## Abstract

Electron beams are often used to treat superficial lesions of the lip, cheek, nose, and ear. Lead is frequently used to block distal structures. It is customary to place an internal bolus of low atomic number in between the tissue and the lead to reduce electron backscatter from the lead. Space for the lead and the internal bolus is quite limited. A previous method for estimating the thickness of the lead plus internal bolus is not self‐consistent and leads to a larger than necessary thickness. A new method is described here to provide a quick, accurate, and self‐consistent estimate of the minimum necessary thickness of the internal bolus and the lead for incident electron beam energies of 4, 6, 8, 9, and 10 MeV as a function of the thickness of the overlying tissue. This method limits the dose enhancement at the tissue/bolus interface due to the underlying lead to 10%. Measurements made with gafchromic film validate this methodology.

## 1 INTRODUCTION

Electron beams are sometimes used to treat superficial lesions of the lip, cheek, nose, and ear for basal and squamous cell carcinoma or keloid formation. In these cases, it is desirable to block the oral and nasal cavity, or for the ear, the scalp, and underlying skull. Lead is generally used for this purpose. The lead causes a dose enhancement at the lead‐tissue interface due to electron backscatter from the lead.^1^ The electron backscatter factor (EBF) is defined as EBF = *D_Pb_*/*D_h_*, where *D_Pb_* is the dose at the interface with the lead present and *D_h_* is the dose at the same depth in a homogeneous medium (no lead). The value of EBF can be as high as 1.5. The EBF is a function of the average electron energy at the lead interface. Low energy electrons are usually used for these treatments, and therefore, the average energy of the electrons at the lead surface is often less than 3–4 MeV after traversing tissue and any external bolus.

**FIGURE 1:**
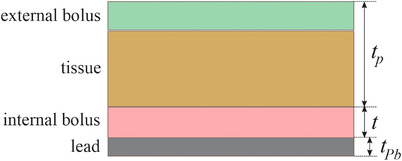
An illustration of the geometry. The variable *t_p_* is the thickness of an externally applied bolus (if present) and the tissue. The variable *t* is the thickness of the internal bolus needed to reduce the backscatter dose enhancement from the lead. The thickness of the lead is *t_Pb_*.

The dose enhancement at the distal tissue surface can be reduced by interposing some low *Z* or tissue equivalent internal bolus material between the lead and the tissue, as illustrated in Figure 1. The lead needs to be covered in any event to avoid tissue contact with this toxic material. The internal bolus absorbs some of the backscatter reducing the dose enhancement. An external bolus may be placed on the skin surface to increase the skin dose. The problem considered here is the determination of the minimum acceptable thickness of the internal bolus and the lead, given the energy of the incident beam and the thickness of the overlying tissue. A quick, self‐consistent method is presented here to find the minimum acceptable bolus plus lead combination that restricts the dose enhancement at the distal tissue surface to 10% above what it would be in the absence of the lead.

A method for calculating the internal bolus and the lead thickness (as referred to by the Task Group 25 report) is described in a widely used textbook by Gibbons as follows:

1) Given the thickness of the overlying tissue, compute the energy of the electrons, *E_m_*, at the distal tissue surface from the average incident electron energy *E*_0_, and the practical range *R_p_*.
2) The EBF is given by Equation (1).
3) Use *E_m_* to calculate the amount of lead needed to block underlying tissue.
4) A graph is provided showing the fraction of the backscatter transmitted upstream in polystyrene (the internal bolus). This graph is consulted to choose a bolus thickness that reduces the backscatter to 10% of its value at the interface with the lead.^1^, ^2^

This calculation algorithm is not self‐consistent. Once the bolus is inserted, the energy of the electrons at the lead surface decreases. This in turn changes the backscatter at the lead surface and the amount of lead and bolus necessary. It would be preferable to have a self‐consistent solution for the necessary thickness of the lead and the thickness of the internal bolus. The algorithm discussed above overestimates the amount of bolus and lead needed. This errs on the side of safety; however, there is very little space in these anatomical locations, and it is desirable to minimize the thickness of the bolus plus lead combination. There is very little space to insert a lead and bolus combination in a patient's nostril, in their mouth, or behind their ear. In addition to this, the formula for the enhancement due to the backscatter (Equation 1) is claimed to be in significant error for energies below 5 MeV at the lead surface and invalid for energies less than 3 MeV. This is based on more recent Monte Carlo (MC) calculations (see below).^3^

Electron backscatter at a tissue/lead interface has been extensively studied both by measurement and MC simulation (see Perez‐Calatayud et al. and references therein).^4^ What has been missing is an easy, accurate, self‐consistent method for putting this information to use in clinical applications.

Klevenhagen et al. have made measurements of the EBF at lead‐polystyrene interfaces using a custom‐made plane parallel ionization chamber for energies of 3–35 MeV at the lead interface.^5^ For incident beam energies below 20 MeV the electron spectra are characteristic of Philips SL75/20 and Philips SL75/10 linacs. Klevenhagen et al. have presented a best‐fit to their experimental data for a lead‐polystyrene interface as follows:

(1) $$\mathrm{EBF}=1+0.735\;\;e^{-0.052E_{m}},$$

where *E_m_* is the average electron energy *at the lead interface*.^4^ The value of EBF can be up to 1.63 for *E_m_* = 3 MeV.

Perez‐Calatayud et al. pointed out that EBF is not well known for energies below 3 MeV.^4^ They have used the MC code GEANT to calculate EBF for energies below 3 MeV with a field size of 20 × 20 cm^2^. They have shown that the EBF *increases* with energy as the energy rises from 0.5 to 1.5 MeV. These authors point out that EBF is difficult to measure because it requires point measurements and the dose enhancement proximal to the lead surface drops rapidly with increasing distance upstream from the interface. Measurements were made to validate the MC calculations. They found that the EBF depends only on the average energy at the interface and not on the initial energy of the electron beam. These authors found that the dependence of the backscatter factor on the distance *t* (in mm) upstream from the lead interface is given by $EBF\left(E_{m},t\right)=EBF\left(E_{m},0\right)e^{-kt}$, where *k* = 0.64 *E_m_* ^−0.63^.

Singh et al. have performed MC calculations of EBF for 6 MeV and 9 MeV electrons beams from a Varian 2100C linac (field size 10 × 10 cm^2^) for a lead‐water interface using the BEAMnrc code.^6^ The authors note that there is a spread in published EBF values below 4 MeV. They cite the following advantages of MC calculations over measurements: (1) “small voxel size (< 1 mm) near the interface and (2) no perturbation due to the physical measurements.” The energy at the interface is as low as 1.0 MeV. The MC calculations were shown to be in good agreement with measurements “except in close vicinity of the lead interface.” This is, however, precisely where accuracy is needed.

Chow and Grigorov have performed MC simulations using EGSnrc simulations for phase space models of a Varian 21 EX for incident energies of 4, 6, 9, 12, and 16 MeV for field size 10 × 10 cm^2^.^7^ These authors have considered the effects of beam obliquity. They claim that the MC code EGS4 underestimates EBF. This problem has supposedly been addressed in EGSnrc.

## 2 METHODS AND MATERIALS

deVries and Marsh have performed MC calculations of the EBF as a function of the electron energy at the surface of the lead.^3^ The mean energy of the electrons at the lead surface ranged from 0.2 to 14.0 MeV. These authors provide a fitting formula for the enhancement in the dose at a distance *t* (water equivalent, in mm) upstream from the interface with the lead, given by:

(2) $$EBF\left(E_{m},t\right)=1+\left(C_{1}e^{-C_{2}\;E_{m}}-C_{3}e^{-C_{4}\;E_{m}}\right)e^{-C_{6}\;t}E_{m}^{C_{5}\;t},$$

where *E_m_* is the mean electron energy in MeV at the lead surface, and *C*_1‐6_ are fitting constants. The values of the constants are: *C*_1_ = 0.936 ± 0.132, *C*_2_ = 0.089 ± 0.012 MeV^−1^, *C*_3_ = 0.602 ± 0.126, *C*_4_ = 0.375 ± 0.065 MeV^−1^, *C*_5_ = 0.130 ± 0.004 mm^−1^ and *C*_6_ = 0.413 ± 0.006 mm^−1^ . The uncertainties are based on a 95% confidence interval. There is a local maximum in EBF(*E_m_*, 0) of about 1.5 at an energy of approximately 3.5 MeV. The fit in Equation (2) reproduces the MC results with an average percentage difference of 1.5% and a maximum percentage difference of 4.9%. Measurements of EBF by deVries and Marsh made with gafchromic EBT2 film show excellent agreement with the calculated values.^3^ These authors state that Equation (1) overestimates the EBF by 11.6% on average over the energy range from 1 to 5 MeV and the maximum overestimation is 31% compared to their MC results.

**FIGURE 2:**
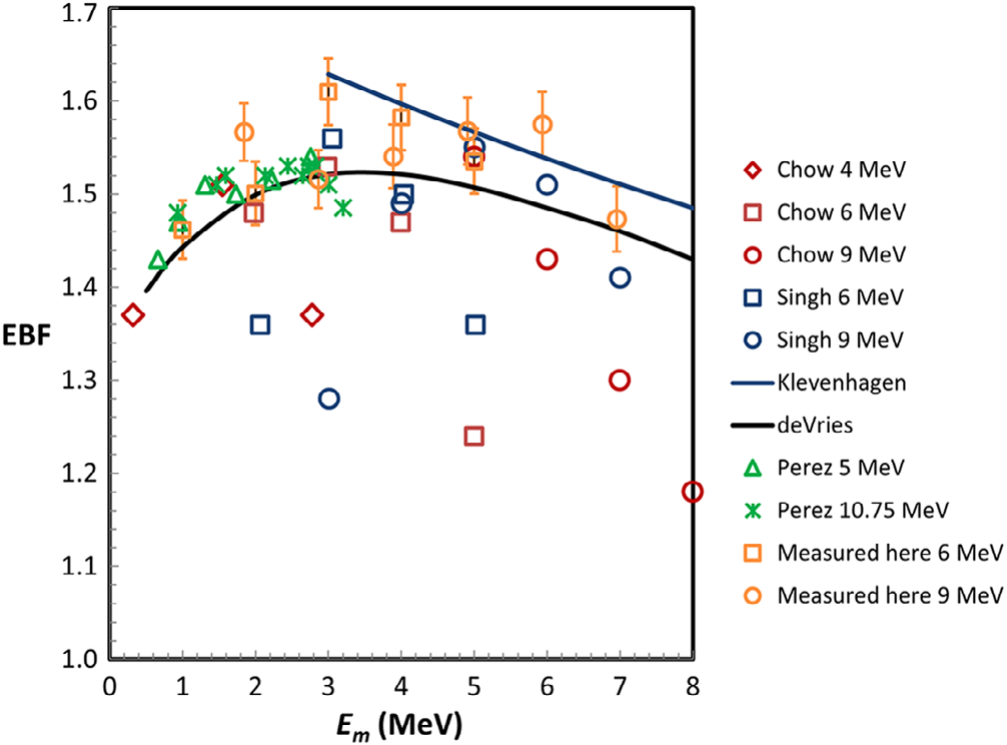
The electron backscatter factor (EBF) calculated or measured by various investigators as a function of the average electron energy at the lead surface. The legend shows the first author of the reference. The energy listed in the legend refers to the average incident energy. The solid curves represent fits to either Monte Carlo or experimental data. There is a mild local maximum for the EBF at an energy of about 3.5 MeV in the deVries data. The measurements made here were made with gafchromic EBT3 film with error bars representing ± 1 standard deviation.

Figure 2 shows various determinations of the EBF as a function of *E_m_* from five different references. It is clear from this figure that there is substantial variation between the different determinations. The deVries et al. values appear to be somewhere in the middle. In addition, the deVries data show excellent agreement with their measurements and have the advantage that an accurate fitting formula (Equation 2) is provided.

The value of *E_m_* depends on the depth of the tissue + bolus interface as follows:

(3) $$E_{m}=E_{0}\left(1-\frac{t+t_{p}}{R_{p}}\right),$$

where *E*_0_ is the average energy of the electrons incident on the patient's skin (or external bolus, in MeV), *t_p_* is the depth of the tissue‐internal‐bolus interface (see Figure 1) and *R_p_* is the practical range of the electron beam. The value of *t_p_* must include the thickness of any *external* bolus placed on the skin surface. The variable *t* can be eliminated from Equation (2) by solving Equation (3) for *t* and substituting back into Equation (2). This results in an equation in terms of a single unknown, *E_m_*. Given a desired value of EBF, the resulting equation can be solved numerically for values of *E_m_* as a function of *E*_0_ and *t_p_*. Equation (3) can then be used to solve for *t*. We have used the Mathematica routine NSolve to solve this transcendental equation.^8^ We have chosen a value of EBF = 1.10, that is, a 10% enhancement in the dose at the tissue‐internal‐bolus interface. In some cases, it may actually be desirable to have a slight enhancement in the dose at this interface if the tissue at the interface is included as part of the target volume. Orthogonal beam incidence has been assumed throughout. No attempt has been made to assess the effects of obliquity.

Values must be chosen for *R_p_* in Equation (3). The rule of thumb that *R_p_* = *E*_0_/2 seems to be remarkably accurate, and we have therefore adopted this expression. Glide‐Hurst *et al*. report on the commissioning of Varian TrueBeam accelerators with *R_p_* = 3.01 ± 0.07 cm for 6 MeV, *R_p_* = 4.43 ± 0.08 cm for 9 MeV, and *R_p_* = 6.11 ± 0.08 cm for 12 MeV.^9^ These measurements were for a 20 × 20 cm^2^ applicator. Narayanasamy et al. report on commissioning of an Elekta Versa HD.^10^ These authors quote *R_p_* = 3.0 cm for 6 MeV, *R_p_* = 4.3 cm for 9 MeV, and *R_p_* = 5.6 cm for 12 MeV. These measurements are for a 10 × 10 cm^2^ applicator.

Once the value of *E_m_* and *t* have been determined for given values of *E*_0_ and *t_p_*, the thickness of the lead *t_Pb_* needed to block distal structures can be computed (in mm) from^2^:

(4) $$t_{Pb}=0.5E_{m},$$

where *E_m_* is in units of MeV.

Equations (2)–(4) have been solved for values of *t* and *t_Pb_* with EBF = 1.10 as a function of the tissue thickness for incident electron energies of 4, 6, 8, 9, and 10 MeV. Uncertainties in the value of the thickness of the bolus, $\sigma_{t}$, have been computed using the formula:

(5) $$\sigma_{t}^{2}=\sum_{i=1}^{6}\sigma_{C_{i}}^{2}{\left(\frac{\partial t}{\partial C_{i}}\right)}^{2},$$

where the values of $\sigma_{C_{i}}$are for 95% confidence intervals specified by deVries and Marsh (values quoted above) for the fitting constants *C_i_*. The partial derivatives in Equation (5) have been evaluated numerically.

In order to validate the results of these calculations, measurements have been made of the EBF in the absence of bolus and with an amount of bolus predicted to reduce the EBF to 110% or less. The measurements have been made using the 6 and 9 MeV beams from an Elekta Versa HD in virtual water using gafchromic EBT3 film. All film samples were from the same lot. A 6 × 6 cm^2^ applicator has been used. The film was calibrated in the 6 MeV beam at a depth of *d_max_* by subjecting samples to 14 different doses ranging from 0 to 500 cGy The optical density (OD) of the film was read with an X‐Rite 301 densitometer with the largest aperture available (3 mm). The densitometer was checked with a calibration optical density “step tablet” prior to film measurements. The OD versus dose data were fit to the following function: $\mathrm{OD}=OD_{0}+\left(OD_{m}-OD_{0}\right)\left(1-e^{-D/D_{1}}\right)$, where OD_0_, OD_m_ and *D*_1_ are fitting parameters. Note that when *D* = 0, OD = OD_0_ and that when D→∞, $\mathrm{OD}\to OD_{m}$. The fit was accomplished using the Mathematica routine “NonlinearModelFit” and the values of the fitting constants are: OD_0_ = 0.241 ± 0.004, OD_m_ = 0.888 ± 0.026 and *D*_1_ = 388 ± 27 cGy. The average root mean square relative error in the fit is 1.6%.

The EBF(*E_m_*, 0) values have been measured at various depths and compared to the predictions of Equation (2) with *t* = 0 and using Equation (3) to compute *E_m_* . The lead used was 6 mm thick. The depths chosen started at 0.5 cm and increased in steps of 0.5 cm up to a depth of 2.5 cm for the 6 MeV beam and 3.5 cm for the 9 MeV beam.

For each depth used to measure EBF, the amount of bolus needed to reduce EBF to 110% or less has been computed. In every case, the calculated amount of bolus, *t*, needed to reduce the EBF(*E_m_*, *t*) to 110% was rounded up to the nearest whole millimeter. The predicted EBF(*E_m_*, *t*) was then recomputed using the rounded value of the bolus thickness. The predicted EBF(*E_m_*, *t*) has then been compared to measured values.

## 3 RESULTS

**FIGURE 3:**
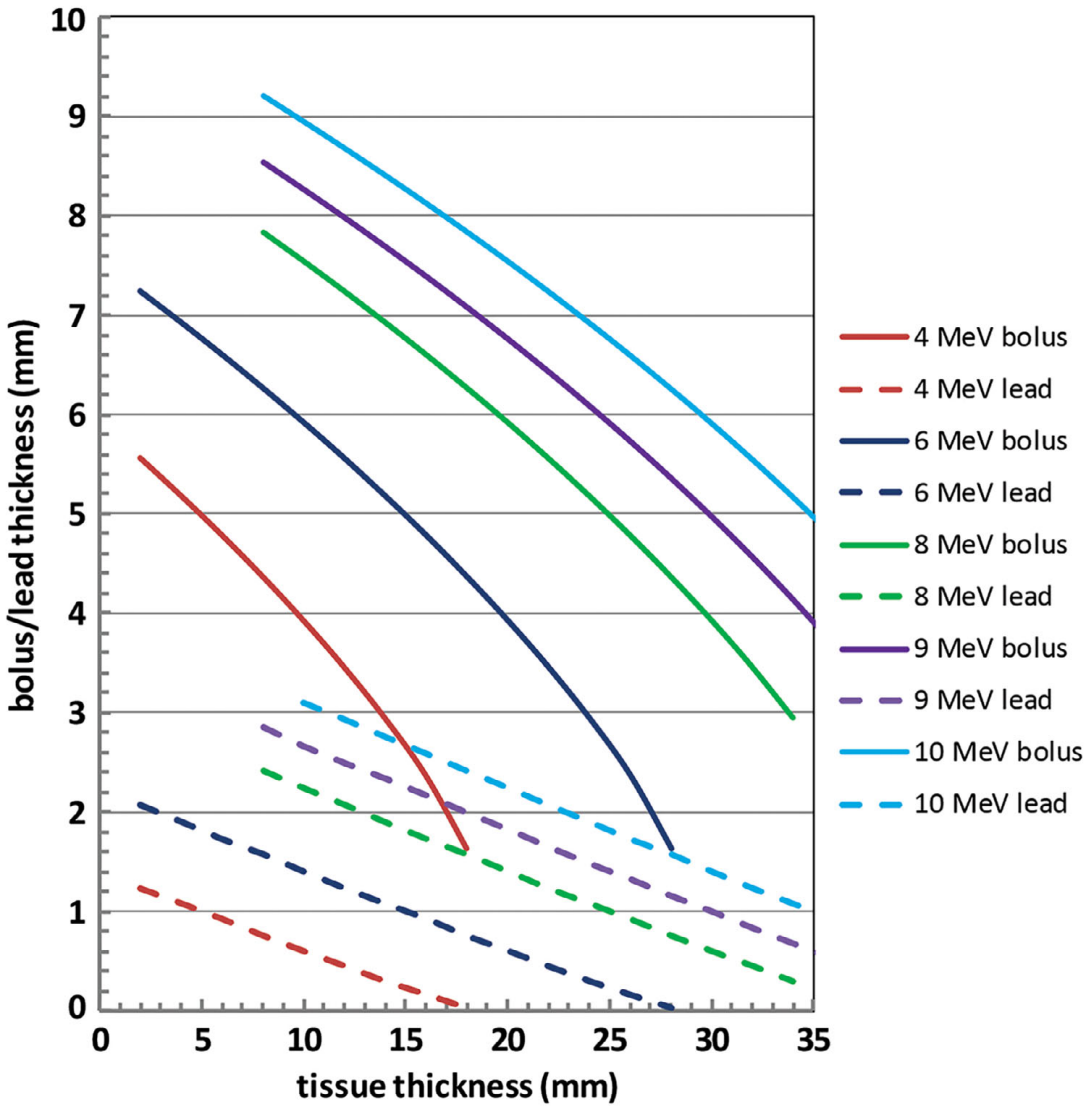
The solid curves show the minimum internal tissue equivalent bolus necessary to reduce the backscatter enhancement in the dose at the tissue/bolus interface so that it is only 10% higher than it would be in the absence of the underlying lead. The dashed curves show the thickness of lead necessary to shield structures distal to the lead. The curves are color coded by energy. Any external bolus must be included in the tissue thickness on the horizontal axis.

Figure 3 shows a set of curves (solid curves) for the required internal bolus thickness as a function of the thickness of the overlying tissue. There is a separate curve for each incident energy of 4, 6, 8, 9, and 10 MeV. Also shown in this same graph is a set of dashed curves that show the necessary lead thickness to shield structures underlying the lead. The thickness of the overlying tissue must include the thickness of any external bolus placed on the skin surface. Given the incident beam energy and the tissue thickness, the user can accurately determine the necessary bolus and lead thickness within a few seconds by consulting Figure 3. As bolus and lead are only available in discrete thicknesses, we advise rounding up to the nearest available thickness. These thicknesses will reduce the enhancement in the dose at the tissue/bolus interface to 10% or less due to the presence of the lead. The uncertainties (95% confidence interval) in the thickness of the internal bolus estimated using Equation (5) are generally between 2% and 3% and the maximum uncertainty is 4.6%.

As an illustration of the methodology described here, let us repeat an example from the textbook by Gibbons.^1^ In this example, a 9 MeV electron beam traverses 20 mm of tissue. Assuming that *R_p_* = 45 mm, the energy of the electrons at the exit surface of the tissue is 5.0 MeV and this will require *t_Pb_* = *E*_0_/2 = 2.5 mm of lead. In order to reduce the backscatter by 90%, roughly 10 mm of bolus is needed, according to Gibbons. According to Figure 3, only about 7 mm of bolus is needed and 2 mm of lead. For an alternative viewpoint, we can take the final parameters computed by Gibbons and calculate the EBF(*E_m_*, *t*) from Equation (2). The total thickness of tissue plus bolus is 30 mm and thus *E_m_* = 3.0 MeV and EBF(3.0, 10) = 1.035.

It is common to use wax as the bolus material. Dental base plate wax (“pink wax”) is commercially available in sheets that are 1.5 mm thick. According to Zhang et al., the electron stopping power of wax is only 6% and 7% higher than that of tissue.^11^

**FIGURE 4:**
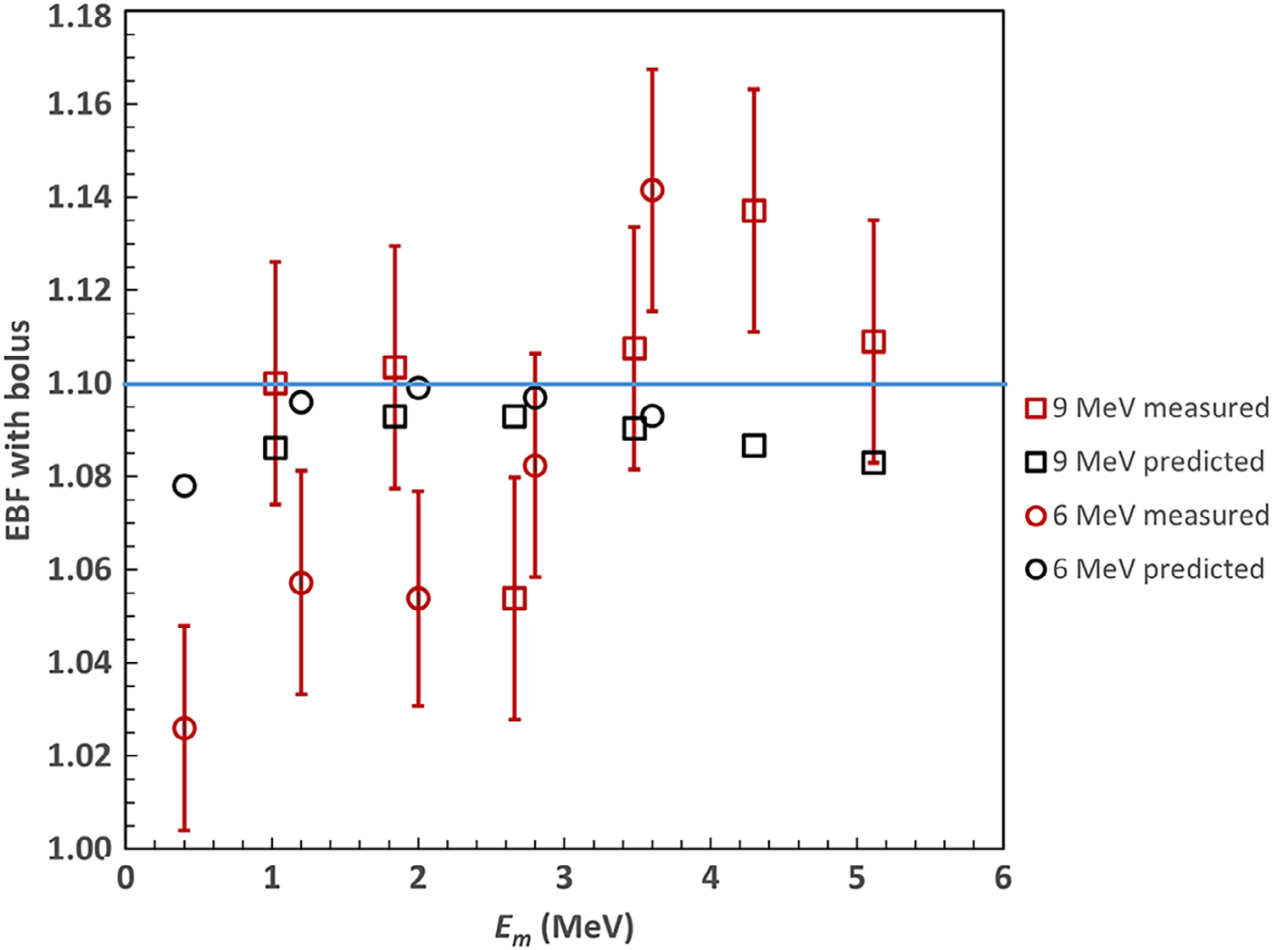
Values of EBF(*E_m_*, *t*) with various values of the bolus thickness *t*, designed to reduce the electron backscatter factor (EBF) to less than 1.10, as a function of the energy at the lead interface for incident beam energies of 6 and 9 MeV. The graph shows predicted values based on Equation (2) and measured values. The error bars on the measured data represent one standard deviation. The EBF ≲1.10 for energies less than 3 MeV and slightly greater than 1.10 for energies above 3 MeV. The maximum measured EBF is 1.14 ± 0.02.

The results of the validation measurements described in section 2 are as follows. Measurements of EBF(*E_m_*,0) are shown in Figure 2 for 6 and 9 MeV incident beams. For energies at the lead interface greater than 3 MeV, the measured EBF values lie between the Klevenhagen (Equation 1) and the DeVries curves (Equation 2). Figure 4 shows a comparison between the predicted EBF(*E_m_*, *t*) plotted as a function of *E_m_* (the energy at the lead surface) with varying amounts of bolus for incident energies 6 and 9 MeV. The bolus thicknesses are as described in the Methods section and were designed to reduce EBF to 1.10 or less. All error bars are ±1 standard deviation.

The average value of the difference between the measured and predicted EBF is 0.3%. On average, the reduction in the dose is greater than 10%, although the reduction appears to be somewhat more than expected below 3 MeV and somewhat less than expected above 3 MeV. This is consistent with the EBF(*E_m_*,0) measurements shown in Figure 2, which are higher than the predicted values for *E_m_* > 3 MeV. The largest measured value of EBF(*E_m_*, *t*) is 1.14 ± 0.02 for 6 MeV at depth 0.5 mm with bolus of a 7 mm. The predicted value is 1.09 ± 0.02. Figure 4 shows that the EBF is reduced to approximately the expected values (i.e., ≲ 1.10) thus validating Figure 3.

## 4 CONCLUSIONS

For clinical electron treatments, a method has been presented to rapidly determine the thickness of lead necessary to shield distal structures and the thickness of tissue equivalent internal bolus needed to reduce the enhancement in the dose due to electron backscatter at the distal tissue surface to 10%. This determination can be made within seconds by consulting Figure 3. This method is self‐consistent, unlike a previously published method, and it takes account of updated backscatter data, including electron energies below 3 MeV at the distal surface. This technique leads to a thinner lead‐plus‐bolus layer than the previously published method. Measurements made with gafchromic EBT3 film validate this methodology.

## AUTHOR CONTRIBUTIONS

None.

## CONFLICT OF INTEREST STATEMENT

The author declares no conflicts of interest.
